# Reviews

**Published:** 1888-04

**Authors:** 


### REVIEWS.

JAHRESBERICHT, DER K. CENTRAL-THIERARZNEI-SCHULE, IN MUNCHEN, 1886-1887. Leipzig, 1888.

This, the history of the work done at the Royal Veterinary School in Munich for the year 1886-87, is a volume of some 150 pages and contains much of interest.

The school is under the directorship of Karl Hahn, who has charge also of the Surgical Department and of the Ambulance Clinic; F. Friedberger, conducts the Internal Clinic and the departments of Special Pathology and Therapeutics; Theodore Kitt succeeds Bollinger, who is now connected with the University, as Professor of General Pathology, Pathological Anatomy, Epidemic Diseases and History of Veterinary Science; J. Feser is Professor of Pharmacology, Toxicology, etc. The other Professors are connected with other institutions as well.

One hundred and thirty-seven students were registered for the winter session, and 107 for the summer session. There were twenty-three candidates for the degree, and of this number twenty successfully passed the examination.

One hundred and fifty-three pathological specimens were added to the museum; 1,642 animals received medical and surgical treatment. Of forty-six animals treated for lobar (croupous) pneumonia, thirty-eight recovered, one improved, six died, and one was under treatment at the time the yearly report was made up. By physical examination in five cases double, in seventeen cases left-sided, and in twenty-three cases right-sided pneumonia was diagnosed.

In the six cases which died, the post-mortem examination showed the following lesions: No. 1, left-sided croupous pneumonia with fibrino-serous pleuritis. Nos. 2 and 3, which in the clinic presented symptoms of severe intermittent colic, and which died thirty-six hours after admission, showed at the post-mortem right-sided croupous pneumonia, pleuritis purulenta hæmorrhagica, lung œdema. No. 4. Pneumonia mortificans of anterior lobe, both lungs and of the middle lobe of the left lung, with left-sided fibrinous pleuritis. No. 5. Death from simple pneumonia, complicated with gastro-enteritis. No. 6. One-sided pneumonia.

INFLUENZA (Pferdestaupe Dickerhoffs). In the spring of 1887 a dealer imported a horse from Belgium. Soon after arrival the animal showed signs of sickness, and two days later (22d April) was brought to the hospital. He had a temperature of 41. 3° C, pulse 48 and respirations 18 per minute, in fact, presented a typical picture of influenza. The case was immediately isolated, the owner informed of the infectious nature of the disease and advised to disinfect the stable. Notwithstanding this caution, other horses were placed in the infected stalls, and on the 3d of May the disease again appeared. Other cases of like mode of infection are cited. In none of the cases was there any lung complications.

Distemper in dogs was treated quite successfully by carbolic water inhalation, combined with antipyretics internally.

There is an interesting article by Prof. Kitt on the protective inoculation against Symtomatic Anthrax, in which, after reviewing the works of Chauveau, Arloing, Cornevin and Thomas on the subject, and comparing them with his own experiments, he arrives at the following conclusions: 1. That a desirable factor to note in the practice of protective inoculation is, that the subcutaneous injection of virus, weakened by submitting it to a temperature of from 85° to 90° C. for six hours, which in its effect is about equal to the 2d vaccine of Arloing, Cornevin and Thomas, though a degree stronger, is quite harmless to cattle, unless given in doses two-fifths and ten times stronger than is necessary to give immunity. 2. That this inoculation material, although it produces in cattle a scarcely perceptible outward effect, or at most only an insignficant local lesion, nevertheless brings about the desired result, as by a single subcutaneous injection complete immunity is obtained, and this holds good for cattle or sheep. 3. That in place of the somewhat inconvenient method of inoculating twice in the tail of the bullock, as advised by Arloing, a single inoculation of his (Kitt's) material is all that is necessary and that for convenience sake it may be done just as well at the shoulder. 4. That through heating, the bacilli spores in the Symtomatic Anthrax muscle, though not fully dead, become so weakened, that in moderate doses the material inoculated into guinea pigs and sheep produces only a local nodule, while in doses of 2-10 cent-grammes it does not possess sufficient virulence to kill the animal. 5. That the above conclusions refer to the Symtomatic Anthrax flesh moistened and heated. The same flesh submitted to dry heat possesses greater virulence, so that of four guinea pigs inoculated two died, and a sheep inoculated developed a severe local swelling. 6. That for protective inoculation the pulverized, dried Symtomatic Anthrax flesh, finely rubbed up in water, is fully as good as the pressed and dried muscle juice, provided that all possible precautions are taken to guard against the evil consequences of the inoculation, *e.g.*, suppuration and abscesses. 7. That not all guinea pigs are rendered immune by protective inoculation. A. W. C.

GIORNALE DI VETERINARIA MILITARE, ANNO 1, NUM 1. UDINE, ITALY, 1888. Editors, F. Regis and L. Baruchello.

This journal, devoted to the care of the cavalry horse, has just entered its career, and the first number gives promise of a periodical of great value to veterinary science, especially in military circles. It will have in view all that relates to the breeding, health and improvement of horses destined for military service.

In the preface, the editor says: "The progress of Science creates specialties, and these are physiological necessities, because the human mind in order to proceed with sure steps, ought to limit its sphere of action, harmonizing its force with the scope which it proposes. This is the great principal subdivision of labor, and the *raison d'etre* of this journal."

American Anthropologist. Published under the direction of the Anthropological Society, Washington, D. C. Vol. 1, No. 1., 1888.

It must be a source of great satisfaction to the Anthropological Society of Washington that it has taken under its auspices a journal which gives such promise in its first number, which has just been issued under the above title. Dr. James C. Welling has an article on the law of Malthus; Col. Seeley, of the Patent Office, one on Time-keeping in Greece and Rome. Dr. Baker on the Human Hand, and Dr. Brinton on the Dialect of Chiapas.

If the succeeding numbers count equally able writers as the above the success of the journal is established beyond doubt. C.

Annali di Agricoltura. Zootecnia, 3 vols. Roma, 1887.

The reports of the Minister of Agriculture to the Italian Government prove that much attention is given to the subject of Animal Industry.

They contain an account of the latest improvements in the care and breeding of horses and cattle, etc., and one volume is devoted entirely to the disease of those animals, and especially of contagious diseases with their attendant bacteria, together with some excellent engravings illustrative of same. C.

Farming World Almanac, 1888, Edinburgh, contains articles on the Prevention and Suppression of Contagious Diseases, by Principal Walley; The Draught Horse: his Feeding and Management, by G. Murray, together with much valuable information for the farmer on sheep-clipping, stock-feeding, bee-keeping, etc.

